# Alterations in iron levels in the locus coeruleus of a transgenic Alzheimer’s disease rat model

**DOI:** 10.1016/j.neulet.2025.138151

**Published:** 2025-02-06

**Authors:** Kayla Aishwarya Bhagaloo, Lei Yu, Elizabeth A. West, Daniel J. Chandler, Natalia Shcherbik

**Affiliations:** a Department of Chemistry and Biochemistry, Rowan University, Glassboro, NJ, 08028, United States; b Department of Cell Biology and Neuroscience, Rowan-Virtua SOM, Stratford, NJ, 08084, United States; c Department of Molecular Biology, Rowan-Virtua SOM, Stratford, NJ, 08084, United States

**Keywords:** Neurodegeneration, The locus coeruleus, Alzheimer’s disease, transgenic rat AD model, Fe, Metals, Fe neurotoxicity, Inductively Coupled Plasma Mass Spectrometry

## Abstract

Iron is essential for brain function, acting as a cofactor for enzymes involved in neurotransmitter synthesis and metabolism. However, dysregulated iron homeostasis is increasingly linked to neurodegenerative diseases, including Alzheimer’s disease (AD). The locus coeruleus (LC), a norepinephrine-producing brainstem nucleus, is among the earliest regions affected in AD, yet its iron dynamics remain poorly understood. This study presents the first comprehensive analysis of iron content in the LC by combining a transgenic AD rat model, precise anatomical isolation, and Inductively Coupled Plasma Mass Spectrometry for high-sensitivity metal quantification. This approach enabled the profiling of iron and zinc concentrations in the LC, uncovering novel insights into iron dysregulation in AD. We observed a significant genotype-specific increase in LC iron levels in TgF344-AD rats compared to wild-type controls. Notably, our findings reveal distinct iron alterations in TgF344-AD rats, suggesting a previously unrecognized role for iron homeostasis in LC dysfunction. These results provide new perspectives on iron dysregulation in AD pathology and its potential as a therapeutic target.

## 1. Introduction

The brain has high levels of iron (Fe), essential for ATP production, macromolecule synthesis, myelin formation, and neurotransmitter production [1]. While insufficient Fe causes neurological impairments, excess Fe promotes neurotoxicity by catalyzing Fenton reactions that generate damaging hydroxyl radicals (•OH), disrupting biomolecules and triggering apoptosis and ferroptosis [2,3]. To prevent oxidative damage, Fe homeostasis is tightly regulated [4]. In the brain, ferritin and neuromelanin sequester excess Fe, particularly in dopamine neurons of the substantia nigra (SN) and norepinephrine neurons of the locus coeruleus (LC) [5,6]. Neurons in both regions require Fe as a co-factor for neurotransmitter synthesis [5]. Aging reduces Fe-buffering capacity, shifting neuromelanin from neuroprotective to neurotoxic and contributing to oxidative damage, supporting the ‘metal-based neurodegeneration’ hypothesis [6].

Fe accumulation in the SN [7], particularly in PD [8,9], correlates with motor dysfunction and is neurotoxic [10,11], as shown in both patients and animal models [12,13]. PD pathology also affects the LC, a key NE-signaling brainstem nucleus. LC is among the earliest regions affected in AD, exhibiting neurofibrillary tangles of hyperphosphorylated Tau years before clinical symptoms emerge [14,15]. This makes the LC a key target for studying Fe dysregulation and its potential contributions to AD pathology. LC degeneration disrupts NE-mediated neuromodulation throughout the nervous system, impairs microglial activity, and contributes to cognitive decline and neurovascular dysfunction, while restoration of NE levels in animal models slows neurodegeneration [16,17].

While tau pathology in the LC is well-documented in aging and AD, information on Fe levels in this region remains scarce, likely due to its small size, deep location, and the need for advanced technologies for precise metal quantification. This study presents the first comprehensive and systematic characterization of Fe concentrations in the LC using a highly sensitive Inductively Coupled Plasma Mass Spectrometry (ICP-MS) technique. This approach was applied to an AD rat model and age-matched wild-type animals, encompassing analyses across young and middle-aged adult cohorts, to elucidate potential age- and disease-associated changes in Fe homeostasis.

## 2. Materials and methods

### 2.1. Animals

The study included 13 TgF344-AD and 12 wild-type (WT) littermates on a Fischer background, ages 2–3 months (n = 13: WT, n = 8 and TgF344-AD, n = 5) and 11–12 months (n = 12: WT, n = 4; TgF344-AD, n = 8). The original breeding pair consisted of male TgF344-AD rats purchased (Rat Resource and Research Center, Columbia, MO) and bred with female Fischer 344 (Envigo, Livermore, CA) to create an in-house breeding colony. TgF344-AD rats were hemizygous for the APPswe/PS1ΔE9 transgene and showed age-dependent pathology and cognitive deficits [18]. Animal care and experimental protocols were approved by the Rowan University IACUC.

### 2.2. Tissue processing and LC collection

Animals were anesthetized with 4 % isoflurane and transcardially perfused with 300 mL of 0.9 % saline, followed by 300 mL of 4 % paraformaldehyde in 0.1 M PBS. Using a Leica CM1860 cryostat, brains were trimmed at the brainstem level to ~ 1.5 mm thickness, containing the full rostrocaudal extent of LC. A 1 mm trephine was used to collect bilateral punches lateral to the fourth ventricle for LC collection. The weight of tissue samples (~1 mg) was determined.

### 2.3. Measurement of Fe and Zn by ICP-MS

Samples were thawed for 2 h at room temperature, digested in 70 % HNO_3_ (110 μL) at 50 °C, and diluted to 2.0 mL with 18 MΩ Direct-Q^®^ deionized water. Fe and Zn (zinc) concentrations were measured using an Agilent 7900 ICP-MS equipped with a He octupole collision cell. Calibration standards (1–100 ppb) were prepared using 2 % HNO_3_.

### 2.4. Statistical data analyses

Statistical analyses were performed using GraphPad Prism 9. Normality was assessed with the Shapiro-Wilk test, and variance homogeneity was evaluated using the Brown-Forsythe test. Two-way ANOVA was used to compare Fe and Zn concentrations in the LC (dependent variables) across genotype groups (WT vs. TgF344-AD, independent variable), followed by Tukey’s post-hoc test where applicable. Statistical significance was set at *P* < 0.05, following standard biological research conventions.

## 3. Results

In this study, we used the transgenic TgF344-AD rat model, which overexpresses mutant human amyloid precursor protein and presenilin, recapitulates hallmark AD pathologies, including age-dependent increases in amyloid-beta plaques, neurofibrillary tangles, microglia activation, neuronal loss, and cognitive deficits, making it a robust platform for detailed investigation of AD pathogenesis. We studied “young adult” (2–3 months old) and “middle-aged adult” (11–12 months old) animal cohorts, each including male and female TgF344-AD rats and age-matched WT Fischer 344 controls. Given that Fe dysregulation is an early pathological hallmark in AD [18] and cognitive and pathological AD-related changes have been documented as early as 10–12 months in the TgF344-AD rat model [18], we considered this timeframe relevant for investigating Fe accumulation in the LC.

LC-containing tissues were isolated and subjected to ICP-MS analysis to quantify trace metal concentrations. ICP-MS enabled simultaneous detection of Fe and Zn at ultra-trace concentrations, with bioactive Zn serving as a biologically relevant internal control. Measuring Fe and Zn in the same samples enhanced accuracy and minimized variability. ICP-MS measurements (counts per second, CPS) were converted to parts per billion (ppb), normalized by LC tissue weight, and expressed as μg/g.

**Fig. 1.**
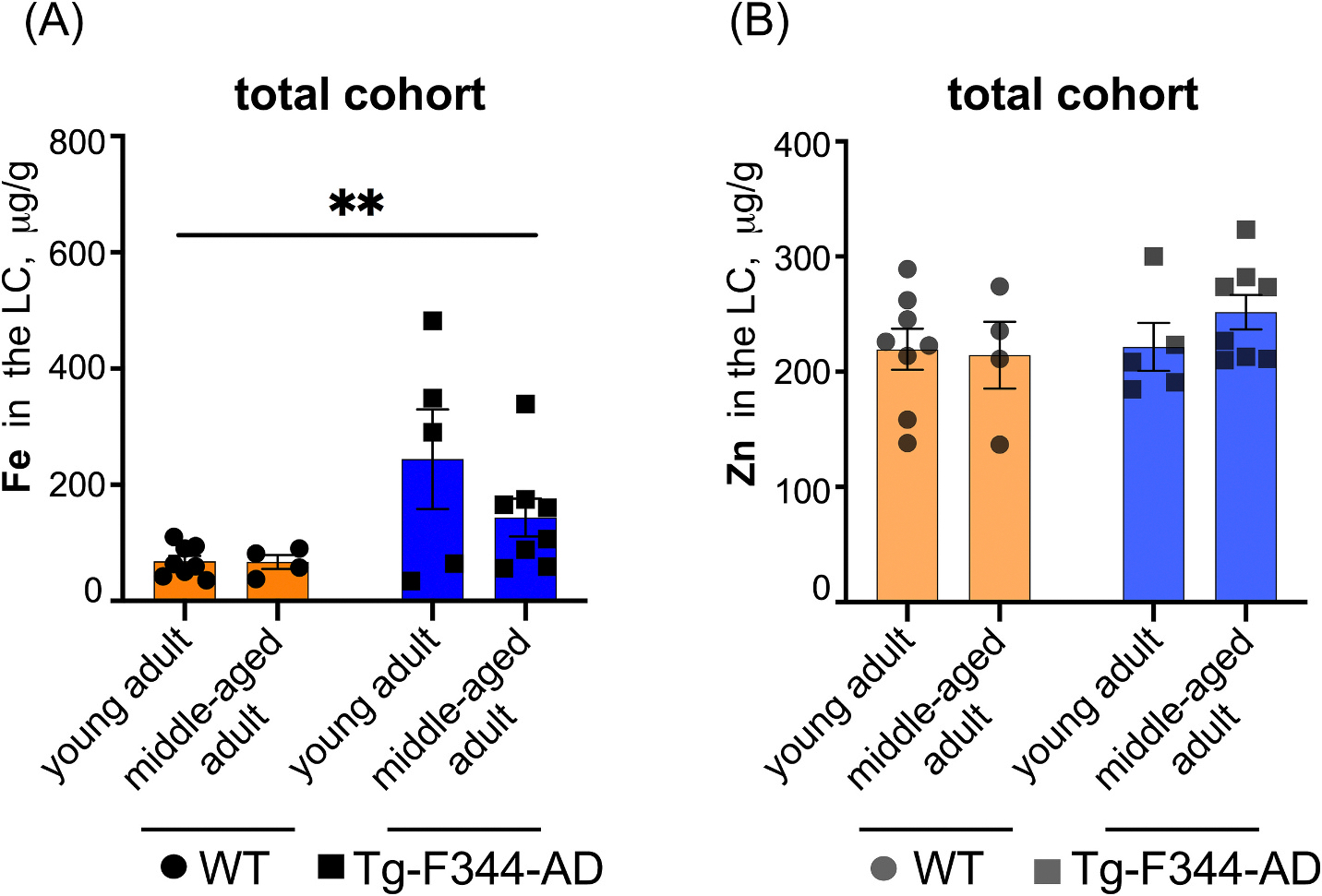
Levels of Fe are elevated in the LC of TgF344-AD rats. A two-way ANOVA with post-hoc Tukey’s multiple comparisons test was used to evaluate Fe (A) and Zn (B) concentrations measured by ICP-MS in the LC of the total animal cohort (males and females) of two genotypes (WT and TgF344-AD) across two age groups, 2–3 months old (young adult) and 10–11 months old (middle-aged adult). Prior to analysis, normality (Shapiro-Wilk test) and variance homogeneity (Brown-Forsythe test) were confirmed, justifying the use of parametric tests. **(A)** Fe concentrations in the LC showed a significant main effect of genotype (F(1, 21) = 9.028, *P* = 0.0068), with TgF344-AD rats exhibiting increased Fe levels compared to WT rats. No significant main effect of age (F(1, 21) = 1.471, *P* = 0.2386) or interaction (F(1, 21) = 1.390, *P* = 0.2516) was observed. **(B)** Zn concentrations in the LC showed no significant effects for genotype (F(1, 21) = 0.9536, *P* = 0.3399), age (F(1, 21) = 0.3870, *P* = 0.5406), or interaction (F(1, 21) = 0.7661, *P* = 0.3913). Bars represent mean ± SEM of Fe or Zn concentrations (μg/g) for each group; (**) *P* < *0.01.*

To analyze ICP-MS measurements of LC-resident Fe and Zn, we first conducted the Shapiro-Wilk test, which confirmed that Fe and Zn concentrations were normally distributed across all groups tested, justifying the use of parametric tests. Brown-Forsythe and Welch’s ANOVA confirmed equal variances (*P* > 0.05), validating the use of two-way ANOVA. Two-way ANOVA revealed significant differences between genotypes, with Fe levels significantly higher in the LC of TgF344-AD rats compared to WT controls (Fig. 1A). However, no significant differences were detected between age groups, and no interaction between genotype and age was observed, indicating that the genotype effect on Fe concentrations was independent of age (Fig. 1A). Post-hoc analysis confirmed that genotype effects were consistent across age groups. In contrast, two-way ANOVA analysis of Zn concentrations detected no significant differences across genotypes, age groups, or their interaction (Fig. 1B).

**Fig. 2.**
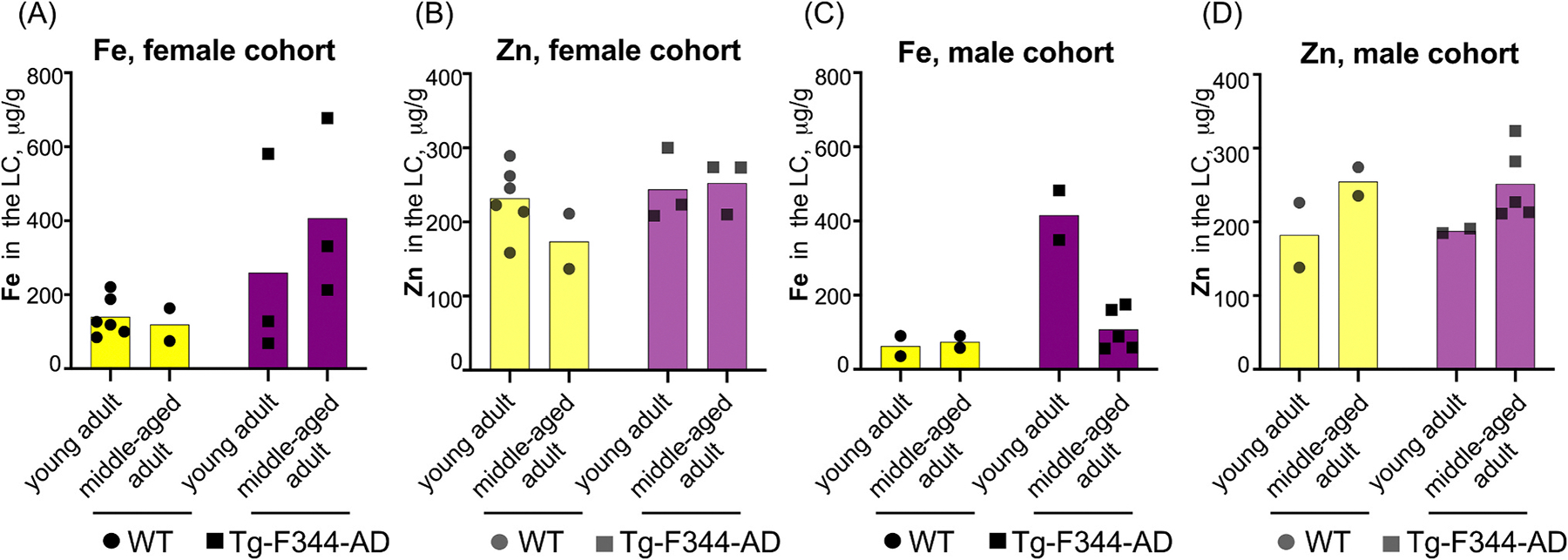
Fe and Zn concentrations in the LC of male and female TgF344-AD and WT rats. **(A-B)**: Fe (A) and Zn (B) concentrations in the LC of female rats across age and genotype groups. **(C-D)**: Fe (C) and Zn (D) concentrations in the LC of male rats across age and genotype groups. Due to limited sample sizes, no statistical analyses were performed. This figure is presented for visual inspection only.

**Fig. 3.**
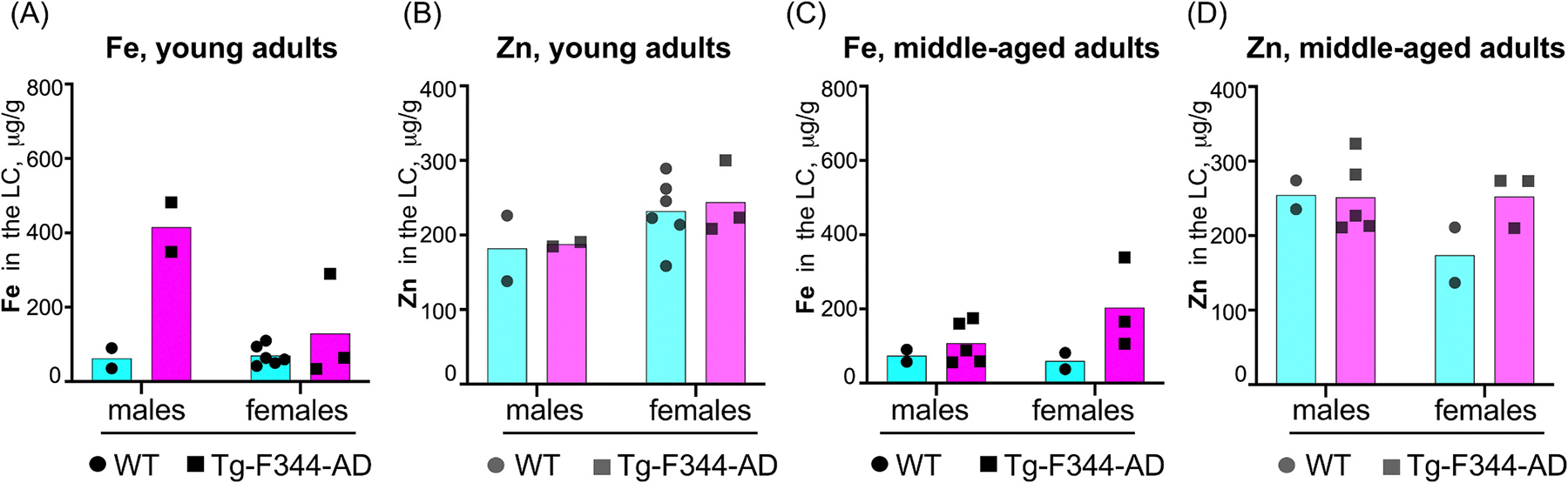
Fe and Zn concentrations in the LC of young and middle-aged adult TgF344-AD and WT rats. **(A-B)**: Fe (A) and Zn (B) concentrations in the LC of young adult animals across genotype and sex groups. **(C-D)**: Fe (C) and Zn (D) concentrations in the LC of middle-aged adult animals across genotype and sex groups. Due to limited sample sizes, no statistical analyses were performed. This figure is presented for visual inspection only.

Given prior reports of sex-dependent differences in AD pathology in both human and rodent models [19,20], we explored Fe and Zn concentrations in the LC separately for male and female cohorts. Due to low sample sizes in individual sex and age groups, no statistical comparisons were conducted. Nevertheless, visual inspection of the data suggests that females did not display significant differences in amounts of Fe and Zn in the LC across genotypes or age (Fig. 2A–B, Fig. 3), while Fe, but not Zn, concentrations were increased in young adult male TgF344-AD rats compared to young adult WT males (Fig. 2C–D, Fig. 3A–B). We also noticed no apparent genotype-dependent differences in Fe and Zn levels for middle-aged males (Fig. 2C–D, Fig. 3C–D). Although our data support the possibility that Fe dysregulation in the LC may be influenced by genotype and age, these trends should be viewed as preliminary and interpreted cautiously, as they require further validation in larger cohorts with increased statistical power to determine whether these trends are biologically meaningful.

## 4. Discussion

Measuring brain Fe levels is critical for understanding its role in normal physiology and the progression of neurodegenerative diseases. Various advanced non-invasive techniques have been employed to identify Fe in specific brain regions in animal models and human patients. Thus, Quantitative Susceptibility Mapping (QSM) and R2* relaxometry have demonstrated increased Fe levels in the SN of aged and PD patients (reviewed in [21]). These findings are corroborated by biochemical methods, including ICP-MS, which have precisely quantified elevated Fe in the same regions in PD-animal models [22]. However, evaluating Fe content in the LC remains technologically challenging, likely due to the nucleus’s small size and deep brain location [15]. As a result, MRI-based studies have struggled to provide conclusive evidence [23]. Using a transgenic AD rat model and the ICP-MS technology, this study provided a sensitive and LC-specific analysis of Fe and Zn, uncovering novel insights into Fe dysregulation in AD.

Although prior research has demonstrated the presence of Fe in the LC neurons, this Fe is typically bound to neuromelanin in humans [24]. However, rats lack neuromelanin unless genetically manipulated, making TgF344-AD particularly advantageous for studying neuromelanin-independent Fe content in the LC. Our findings revealed significantly elevated Fe in the LC of transgenic animals compared to WT, aligning with studies linking Fe accumulation to AD pathology and suggesting a mechanism independent of neuromelanin.

Furthermore, the absence of age-dependent Fe accumulation in WT vs. TgF344-AD cohorts within the sex-mixed analysis (Fig. 1A) suggests that aging does not play a critical role in Fe accumulation in the LC when males and females are analyzed together. However, sex-specific differences in Fe accumulation may exist. For the male cohort, but not females, young adult TgF344-AD males exhibited higher Fe levels in the LC than middle-aged adult males (Fig. 2A–C and Fig. 3), pointing to a potential shift in Fe regulation during disease progression specific to males. This observation aligns with studies demonstrating that males experience faster cognitive decline and more severe pathology [25].

Given that LC-resident Zn concentrations remained consistent (Fig. 2D), our data suggest that Fe-specific mechanisms, rather than generalized neuronal loss, may contribute to these observations. Nevertheless, simultaneous histological and ICP-MS analyses of the LC would be ideal to provide a direct correlation between the potential loss of LC neurons in advanced disease states and Fe content.

While our study provides critical insights into Fe dysregulation in the LC in the total cohort of animals, it is essential to emphasize that our sex- and age-dependent data are underpowered for robust statistical comparisons due to limited sample availability, preventing firm conclusions. These findings should, therefore, be considered preliminary.

Future studies with larger cohorts of both males and females, spanning a broader age range (young 2–3 months, middle-aged 10–11 months, and older 18–24 months adult cohorts), will be needed to comprehensively characterize Fe homeostasis in AD pathology. A fully powered study including both sexes is essential to better understand Fe dysregulation in disease progression and determine whether early Fe accumulation in males contributes to LC dysfunction in AD.

## Data Availability

Data will be made available on request.

## Significance statement

This study provides the first comprehensive analysis of metal content in the locus coeruleus, revealing iron accumulation in the transgenic AD rat model, unchanged zinc levels, and potential sex-specific differences in iron homeostasis.
